# Temperature dependence of piezo- and ferroelectricity in ultrathin P(VDF–TrFE) films†

**DOI:** 10.1039/c8ra05648j

**Published:** 2018-08-16

**Authors:** Jun Qian, Sai Jiang, Qijing Wang, Chengdong Yang, Yiwei Duan, Hengyuan Wang, Jianhang Guo, Yi Shi, Yun Li

**Affiliations:** National Laboratory of Solid-State Microstructures, School of Electronic Science and Engineering, Collaborative Innovation Center of Advanced Microstructures, Nanjing University Nanjing 210093 P. R. China yli@nju.edu.cn

## Abstract

The polymer poly(vinylidene fluoride-*co*-trifluoroethylene) (P(VDF–TrFE)) is highly desirable for piezoelectric and ferroelectric functional applications owing to its considerable electromechanical activity and reliable electrical polarization. However, a clear understanding of the effect of the thermal annealing on the electromechanical behavior and polarization nature of ultrathin crystalline P(VDF–TrFE) films is severely lacking. Here we report the thermally induced structural reorganization, and piezo- and ferroelectric features in the ultrathin P(VDF–TrFE) films. On applying a 40 °C annealing treatment, the polarization-patterned electrostrictive strain reaches the highest value of ∼53.7 pm. Besides, the ultrathin film exhibits a highly ordered antiparallel dipole alignment, the highest local piezoelectric activity, and an improved polarization relaxation time. The optimum film properties are achieved owing to a high degree of polymer chains oriented parallel to the substrate plane. Our results can reveal a promising avenue for nano-electro-mechanical and nano-ferroelectric electronic applications using ultrathin P(VDF–TrFE) films.

## Abstract 2

The electromechanical activity and polarization nature of the quasi-2D ultrathin polycrystalline P(VDF–TrFE) were clearly demonstrated, revealing a promising avenue for nano-electro-mechanical and nano-ferroelectric applications using ultrathin P(VDF–TrFE) films.

## Graphical abstract

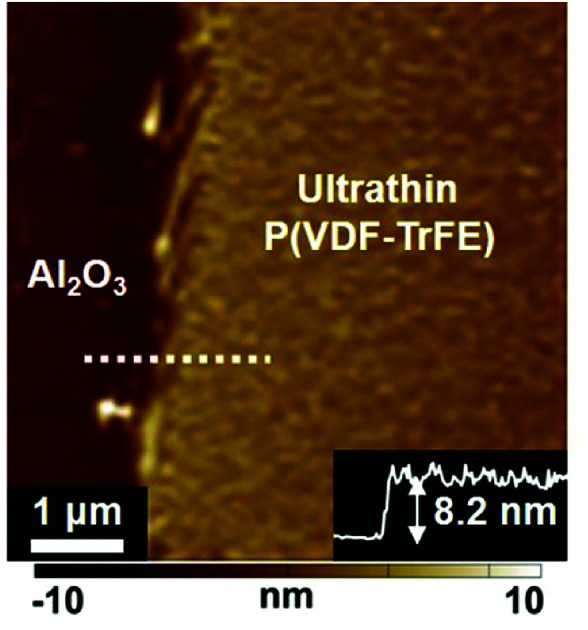

## Introduction

The discovery of piezoelectricity and ferroelectricity in poly(vinylidene fluoride-*co*-trifluoroethylene) (P(VDF–TrFE)) offers an advantageous opportunity to electrically manipulate its functional properties.^1,2^ The molecular ferroelectric polymer P(VDF–TrFE) can be light weight, highly stretchable, bio-compatible, capable of adaptivity, and stimuli-sensitive, which guarantee it a steady rise towards personal and portable electronics.^3–7^ Particularly, thanks to an exceptional electromechanical activity and large permanent electrical polarization, P(VDF–TrFE) is worth exploring as a competitive candidate in sensing systems, transducer devices, and non-volatile memory cells.^8–18^ Note that, a severe bottleneck for practical applications of P(VDF–TrFE) in advanced electronic technologies is the achievement of a reasonable mechanical and electrical performance; also, to decrease the film thickness is greatly important to alleviate the power consumption and unreliable repeatability of functional elements caused by the high-voltage operation.^19–22^ However, traditional approaches such as electrospinning, Langmuir–Schaefer (LS) deposition, nanoconfinement, and addition of crystallization-inducing agents suffer from limited applicability to enhance the film properties due to a degradation of ultrathin film quality.^11,23–27^ Furthermore, for an ultrathin polycrystalline P(VDF–TrFE) film under a special constrained geometry, the film properties (*e.g.*, film morphology, crystalline conformation, and lattice orientation) should be significantly altered by a post thermal-annealing treatment, which has rarely been reported.^22,28–30^ Besides, to elucidate the temperature-dependent nanoscale piezo- and ferroelectric properties of P(VDF–TrFE) is also favourable for enlarging its application basis available to emerging nanoelectronics.^17,31^ Recently, our work has revealed that the ultrathin crystalline P(VDF–TrFE) films with highly morphological uniformity can be prepared *via* a solution-based technique.^32^ Consequently, it is of key importance to study the piezo- and ferroelectric properties of ultrathin crystalline P(VDF–TrFE) films upon the thermal annealing treatment.

In this study, we present a clear evaluation of the electromechanical response and polarization feature with different thermal annealing temperatures in the ultrathin P(VDF–TrFE) films. In particular, the optimum piezo- and ferroelectric characteristics can be achieved at 40 °C. The polarization-defined relative morphological deformation of the ultrathin film can reach up to ∼53.7 pm and the molecular electric dipoles adopt a highly-ordered antiparallel packing. Besides, the ultrathin films exhibit the largest local piezoelectric coefficient *d*_*zz*_ and polarization relaxation time. The enhanced film properties are ascribed to a high polymer chain orientation degree parallel relative to the substrate. Our results can provide insightful understanding of the intercorrelation among the thermal crystallization and film properties of crystalline P(VDF–TrFE) films at the nanoscale. Such ultrathin P(VDF–TrFE) films will also motivate potential functional applications ranging from self-powered nano-electro-mechanical systems (NEMS), to next-generation bio-implanted detectors and ultrahigh-density data storage.

## Experimental

### Fabrication of quasi-2D ultrathin P(VDF–TrFE) films

Silicon (Si) wafers were used as the starting substrates. Subsequently, 10 nm Al_2_O_3_ thin film was deposited on the pre-cleaned Si substrate by thermal atomic layer deposition (TALD). TALD was conducted in a commercial flow-type hot-wall reactor (Picosun SUNALE R-200B, Finland). P(VDF–TrFE) (the molar ratio between VDF and TrFE was 70 : 30) was received from Solvay, Inc., France. It was dissolved in a DMF solution (2.5 wt%) with a small amount of antisolvent *p*-anisaldehyde (2 mg ml^−1^). The solution was drop-cast onto an Al_2_O_3_/Si substrate. And then, a mechanical pump generated airflow that dragged the droplet to rapidly move on the substrate. The ultrathin crystalline P(VDF–TrFE) film was formed within several seconds. Four kinds of samples (the first kind of films were prepared without any post-annealing treatment, whereas the other samples were annealed at different temperatures, from 40 °C up to 60 °C on a hot plate for 10 min) were fabricated in a nitrogen-filled glove box.

### Characterizations of quasi-2D ultrathin P(VDF–TrFE) films

The step height and surface topography of our ultrathin crystalline P(VDF–TrFE) films were characterized by using atomic force microscopy (AFM, Asylum Research Cypher scanning probe microscopy, Oxford Instruments, Shanghai, China) in tapping mode. The crystalline phase was identified using a NanofindeNr FLEX Raman confocal Microscope and a Rigaku Smartlab XRD equipment. For the GIXRD characterization, A Rigaku Smartlab XRD instrument with 3 kW X-ray power was used. The incident angle is fixed at an angle of 0.05° during the measurement. For 2*θ* scan, incident X-ray and ultrathin films were not moving, and the detector moved anticlockwise. For the characterization of the piezoelectric activity and ferroelectric nature, resonant-enhanced piezoresponse force microscopy (PFM) mode was performed. Typical in-contact resonant frequency range for an AC voltage was 320–350 kHz with an amplitude of 0.8 V (peak-to-peak). Commercial silicon tips coated with chromium/platinum–iridium (Cr/Pt–Ir, Nanosensors PPP-EFM) were positioned at a selected area on the bare P(VDF–TrFE) film surface. The bias voltages were applied to the PFM tip, and the bottom Si substrate was grounded. For the measurement of local piezoelectric coefficient *d*_*zz*_, the displacement amplitudes of the piezoresponse of the ultrathin crystalline P(VDF–TrFE) films were measured under different external voltages. The local *d*_*zz*_ values of the ultrathin films were extracted as the slope of the linear fitting of the curve. All the PFM measurements were performed under room temperature.

## Results and discussion

**Fig. 1:**
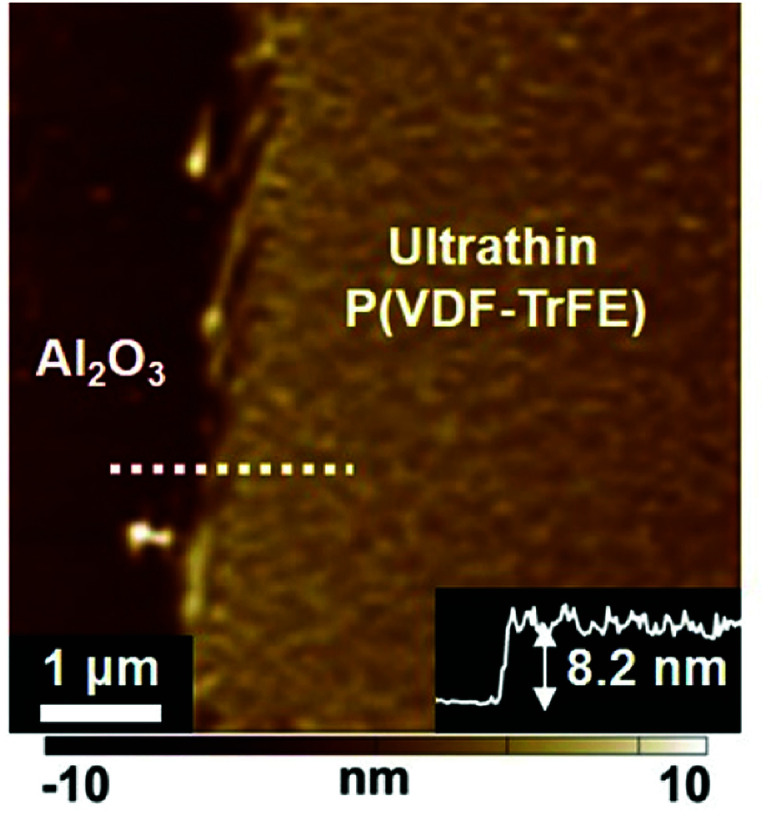
The AFM height image of the quasi-2D ultrathin P(VDF–TrFE) film. The height profile is along the white dotted line shown in the image.

P(VDF–TrFE) was dissolved in a mixture solvent with *N*,*N*-dimethylformamide (DMF) and immiscible *p*-anisaldehyde that served as a major solvent and an antisolvent, respectively. We previously showed that an antisolvent-assisted-crystallization technique can be used to fabricate ultrathin crystalline P(VDF–TrFE) films.^32^ 10 nm-thick Al_2_O_3_ thin film was deposited on the Si substrate by TALD and exhibited a ultrasmoothness at the atomic level (Fig. S1†). Then, P(VDF–TrFE) films were prepared on the Al_2_O_3_/Si substrates in a glove box at room temperature. As shown in Fig. 1, the thickness of the P(VDF–TrFE) film is approximately 8.2 nm. In addition, the ultrathin film exhibits good continuity over a large area. Thus, our crystalline P(VDF–TrFE) film shows a typical quasi-2D ultrathin feature with the smooth and neat uniformity (Fig. S2†). Besides, an attractive point is that the crystals can be prepared on arbitrary substrates, such as Si, Au, Pt, and semiconducting In_2_O_3_ (Fig. S3†); also, the film thickness can be easily controlled by tuning the P(VDF–TrFE) concentration in the solution (Fig. S4†).

**Fig. 2:**
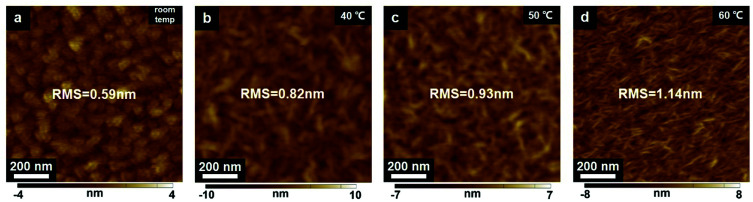
(a–d) AFM morphological images of ultrathin P(VDF–TrFE) films with different annealing temperatures.

**Fig. 3:**
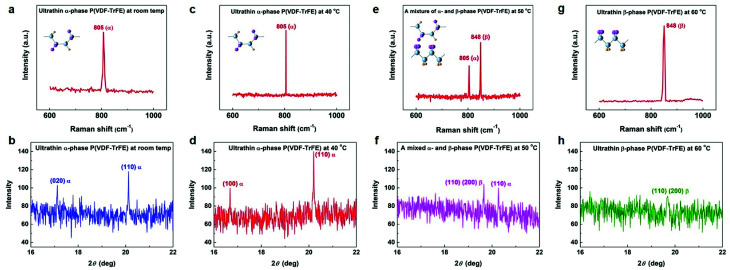
Raman spectrums (a, c, e and g) and GIXRD diffraction patterns (b, d, f and h) of ultrathin P(VDF–TrFE) films with different annealing temperatures. Insets represent the molecular conformations α- and β-P(VDF–TrFE).

To assess temperature-dependent crystalline properties, four kinds of ultrathin films (as-grown films under room temperature, and the other coated films were post-annealed at 40 °C, 50 °C, and 60 °C for 10 min, respectively) were prepared on the Al_2_O_3_/Si substrates. The morphological and structural images of ultrathin crystalline P(VDF–TrFE) films at four temperature levels are shown in Fig. 2 and 3. At room temperature, the polycrystalline P(VDF–TrFE) surface exhibits a small root-mean-square (RMS) roughness of 0.59 nm, and the lamellae grains of P(VDF–TrFE) are clearly visible with a typical lateral size of ∼100 nm (Fig. 2a). The Raman spectra of the P(VDF–TrFE) film presents a sharp peak at 805 cm^−1^, corresponding to the crystallographic characteristic of the α-phase (Fig. 3a).^19^ Additionally, the achievement of the crystalline α-phase was also confirmed by the grazing-incident X-ray diffraction (GI-XRD) characterization (Fig. 3b).^10,33^ When the ultrathin P(VDF–TrFE) film is annealed at 40 °C, a surface RMS roughness of 0.82 nm can be observed (Fig. 2b). As shown in Fig. 3c and d, the remarkable Raman peak and GI-XRD characteristic pattern can be obtained, which are assigned to the crystalline α-phase. Thus, our ultrathin α-P(VDF–TrFE) is capable of maintaining its structural stability under a 40 °C annealing treatment. The α-phase P(VDF–TrFE) is stable due to the specific structures such as head-to-head defects and chain ends. When the P(VDF–TrFE) film is annealed at an elevated temperature of 50 °C, the surface roughness increases up to 0.93 nm, as illustrated in Fig. 3c. Moreover, the Raman peaks obtained at 805 cm^−1^ and 848 cm^−1^ are corresponding to the characteristic of α- and β-phases, respectively (Fig. 3e). And the GI-XRD diffractogram can also confirm this crystalline structure in our ultrathin P(VDF–TrFE) films. These results indicate that a portion of α-phase still remains and an α- to β-phase transformation occurs in our ultrathin film at 50 °C. It is because that the presence of the TrFE monomers of P(VDF–TrFE) destabilized the α structure. Thus, P(VDF–TrFE) ultrathin films appeared to contain two phases. As further increasing the annealing temperature to 60 °C, the P(VDF–TrFE) film exhibits the surface roughness of 1.14 nm (Fig. 2d). In addition, a clear Raman peak at 848 cm^−1^ and a GI-XRD diffraction signal at 2*θ* of 19.7°, which are the characteristic of the crystalline β-phase, are expected to present (Fig. 3g and h). It is in good agreement with our previous study, manifesting that the α- to β-phase transformation is complete at 60 °C.^32^

As stated above, a thermally-induced α- to β-phase transformation behavior accompanying the change of the lamellar morphology is obtained in quasi-2D ultrathin crystalline P(VDF–TrFE) films. During thermal crystallization, lamellae structure of crystallites changes from a spherulitic to a fibrillar, and the metastable α-chain reorganizes to form the most thermodynamically stable β-chain. The reader is referred to another theoretical paper for understanding the molecular geometry of α- to β-chain P(VDF–TrFE).^34^ Additionally, when the post-annealing treatment on the ultrathin film was conducted at 135 °C, which was above the curie transition but below the melting temperature, the resulting crystals are still composed of a β-phase without any conformational change (Fig. S5†). Besides, the morphology of lamellae grains is similar to the one observed in spin-cast films.^35^ This result clearly demonstrated the structural stability of quasi-2D ultrathin crystalline P(VDF–TrFE) films under high temperature, which is critical for realistic large-scale integration in the near future.

**Fig. 4:**
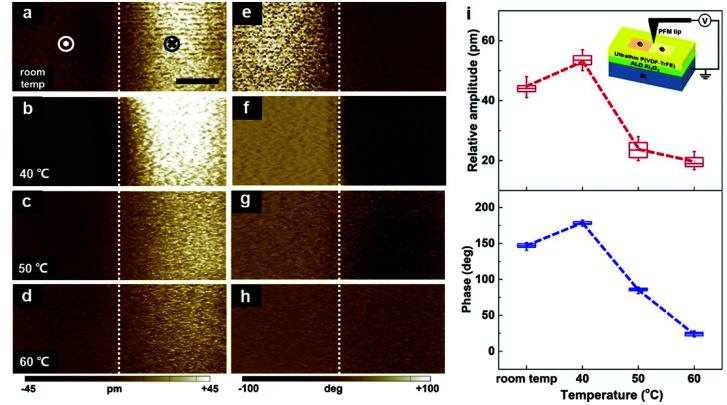
Temperature-dependent domain piezo- and ferroelectric properties of ultrathin P(VDF–TrFE) films. (a–d) The out-of-plane PFM amplitude images at different annealing temperatures. (e–h) The out-of-plane PFM phase images at different annealing temperatures. All PFM images were obtained over the polarization-patterned areas, and the white dotted lines were the boundaries between neighboring polarized areas. Scale bar: 2 μm. (i) The evolution of the relative amplitude and phase contrast with the annealing temperature. The inset was the schematic of the PFM experimental setup for demonstrating the domain piezo- and ferroelectric properties in the ultrathin crystalline P(VDF–TrFE) films.

High-sensitivity piezoresponse force microscopy (PFM) was used to probe the piezo- and ferroelectric behaviours.^36^ We studied the electromechanical response and polarization nature by writing rectangular areas with downward and up polarization orientations and then imaged the pre-written areas. An ultrathin crystalline P(VDF–TrFE) film was written by a biased PFM tip. The left- and right-hand part of a scanning domain was poled at different voltages (−9 V and +9 V) with a defined upward and a downward polarization state, respectively. Followed by a PFM tip scans covering the whole pre-poled regions. Fig. 4a–h shows the PFM images obtained for the four samples at different temperature levels. The changing tendency of the relative amplitudes and phase contrasts between two different polarization states are illustrated in Fig. 4i. For all samples, the amplitude images exhibit an evident contrast for binary polarization states (Fig. 4a–d), which is a typical signature of the negative piezoelectric effect of the ferroelectric polymer P(VDF–TrFE).^37^ The bright (dark) PFM phase image for polarization upward (downward) is in good agreement with the negative piezoelectricity of P(VDF–TrFE). Particularly, a striking electrical polarization-biased electrostrictive strain of ∼53.7 pm can be obtained in a 40 °C-annealed film. This electromechanical activity has been proven to root from the polar α-P(VDF–TrFE) in our previous work.^32^ In addition, the ferroelectric features can be verified by the PFM phase images (Fig. 4e–h), which indicate that a reliable ferroelectricity with the homogeneous polarization can be sustained down to a few nanometers scale in P(VDF–TrFE). The electrical polarization is stabilized by van der Waals forces between polymer chains.^38^ Notably, a clear 180° phase contrast in a 40 °C-annealed ultrathin film reveals a highly-ordered dipole antiparallel orientation, and smaller phase contrasts in other samples are mainly driven by a polarization reversal constraint effect.^22^ These observations point to 40 °C-annealed samples with the superior piezoelectric deformation and excellent polarization arrangement.

**Fig. 5:**
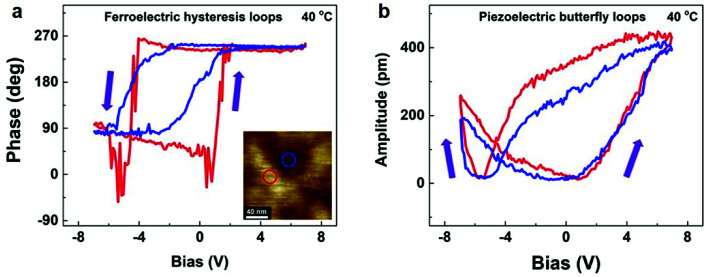
Local ferro- (a) and piezoelectric hysteretic loops (b) of different regions in an ultrathin P(VDF–TrFE) film. The red and blue hollow circle shown in (a) represent the grain and intergrain region, respectively. The arrows indicate the sweep direction.

The film morphology of P(VDF–TrFE) is generally considered to have a profound impact on the electromechanical response and polarization behaviours.^8,19,39,40^ Note that polycrystalline P(VDF–TrFE) films are generally composed of a composite microstructure with coexisting crystalline and amorphous parts.^37,41^ Thus, the piezo- and ferroelectricity inevitably exhibit an average effect incorporating the grain and intergrain regions.^37,42^ However, the local piezo- and ferroelectricity of nanosized P(VDF–TrFE), which are associated with different film regions, are still far from being clear. Therefore, establishing a precise correlation between the film regions and local piezo- and ferroelectric properties is of key significance to control the film morphology towards further enhancing the mechanical and electrical performance of P(VDF–TrFE). Considering that the grain size (∼100 nm) is larger than the tip resolution (∼50 nm), we can separately study local PFM characteristics on the grain and intergrain regions in quasi-2D ultrathin P(VDF–TrFE) films. As shown in the inset of Fig. 5a, the grain and intergrain region are marked with a red and a blue hollow circle, respectively.

To explore local ferroelectric features, we first performed phase *versus* voltage measurements on two different regions of 40 °C-annealed ultrathin film. A conductive PFM tip was directly in contact with our pristine P(VDF–TrFE) film surface (in a ∼50 nm-diameter area) connected to an electric source. Local PFM hysteresis loops were measured at selected locations. As shown in Fig. 5a, on the grain region, the typical ferroelectric characteristic is very clear with a nearly symmetric rectangular-like hysteresis loop, in which a 180° phase contrast is observed. Meanwhile, the grain region presents the coercive voltages (*V*_c_) of +1.4 V and −4.2 V, at which ferroelectric polarization reversal occurs. Besides, we also obtained a distinct ferroelectric hysteresis shape on the intergrain region, in which the widths of the coercive voltages broadened, ranging from −1.0 V to +1.2 V and from −5.0 V to −3.0 V. In addition, the polarization switching and electromechanical response should simultaneously occur at a measured region. Thus, the local piezoelectric features can be characterized *via* the amplitude *versus* voltage measurements. Fig. 5b shows that the classical butterfly-shaped piezoelectric hysteresis loops can be obtained for both the grain and intergrain regions. And the coercive voltages for two different regions are similar to the observations of the ferroelectric hysteretic behaviours, as depicted by the minima of the amplitude–bias curves. Thus, to downscale the ferroelectric film thickness is helpful for greatly decreasing the operation voltage to drive the molecular dipole rotation around the polymer chain plane. Moreover, our local measurements performed at various locations (>20 areas) show reliable reproducibility and high uniformity of the observed results. We also characterized local ferro- and piezoelectric hysteretic loops for other three temperatures (Fig. S6†). Therefore, the grain and intergrain regions in our ultrathin P(VDF–TrFE) films can both exhibit clear signatures of the ferroelectric polarization states and piezoelectric deformation behaviours.

**Fig. 6:**
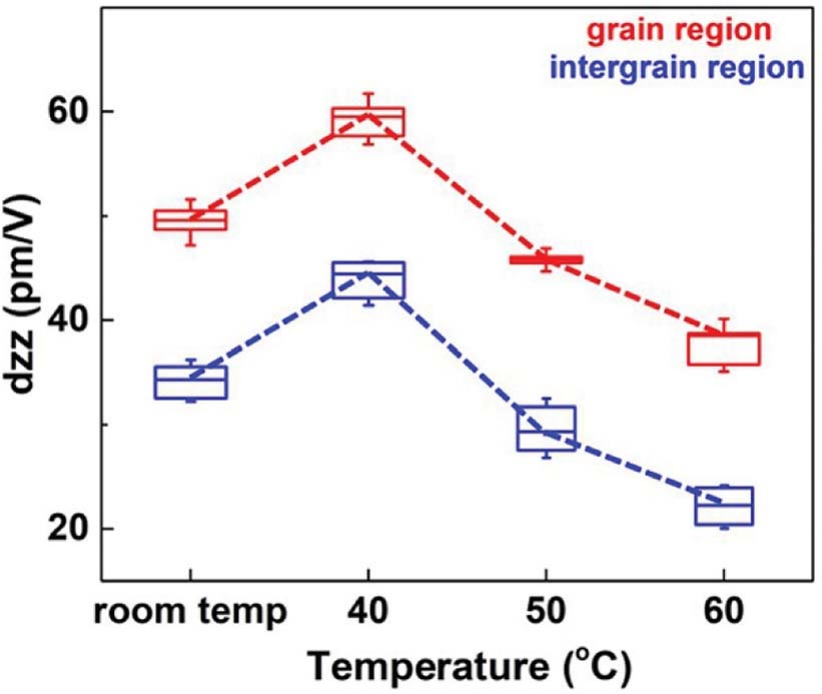
The evolution of the local piezoelectric coefficients *d*_*zz*_ with different temperatures for both the grain and intergrain regions. It illustrates that the maximum *d*_*zz*_ is obtained at 40 °C, and the identical changing tendency with the temperature for two different regions can also be observed.

The local piezoelectric coefficient *d*_*zz*_, which represents the linear response of the piezoelectric amplitude displacement to the external electrical voltage, can provide a quantitative insight into the local electromechanical activity.^43^ Thus, we implemented the measurement of *d*_*zz*_ on both the grain and intergrain regions for four temperatures (room temperature, 40 °C, 50 °C, and 60 °C). Besides, *d*_*zz*_ values were measured by applying quasi-static poling voltages. As illustrated in Fig. 6, under room temperature, the average *d*_*zz*_ value of the grain region is −49.6 pm V^−1^, and the measured *d*_*zz*_ of the intergrain region can also reach −34.7 pm V^−1^. A pronounced variation can be observed at 40 °C, the *d*_*zz*_ values of the grain and intergrain region dramatically increase to −59.3 pm V^−1^ and −45.8 pm V^−1^, respectively. Moreover, it is clear that the evolution of *d*_*zz*_ values exhibit a notable decreasing tendency with the increase of the post-annealing temperatures. In addition, the *d*_*zz*_ of the grain region (intergrain region) decreases from −47.8 pm V^−1^ (−29.4 pm V^−1^) to −41.2 pm V^−1^ (−22.7 pm V^−1^) by increasing the annealing temperature from 50 °C to 60 °C. From these local piezoresponse measurements for both the grain and intergrain regions, the largest electrostriction in our ultrathin crystalline P(VDF–TrFE) films can be obtained with a 40 °C post-annealing treatment.

**Fig. 7:**
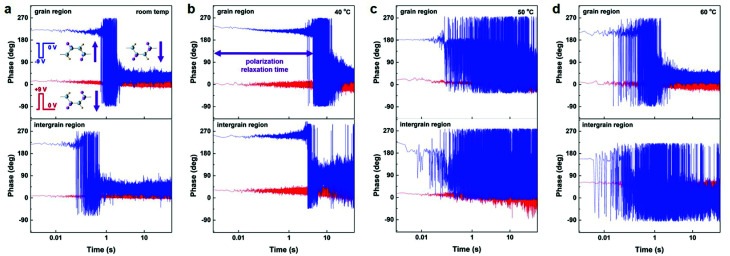
The ferroelectric polarization nature after removal of the external poling field for both the grain and intergrain regions at four temperature levels. The phase of two ferroelectric polarization orientations is presented as a function of the time. Insets are the molecular structures and polarization states of P(VDF–TrFE).

The local ferroelectric properties for both the grain and intergrain regions were further evaluated *via* the detection of binary ferroelectric polarization states (that is, +P and −P), as described previously.^32^ The PFM phase images of ferroelectric polarization states as a function of the delay time after removing the poling voltages are depicted in Fig. 7. For both the grain and intergrain regions at four temperature levels, the downward polarization orientation is defined by poling the specific point on the ultrathin film with a positive voltage. And by changing the polarity of the PFM tip-generated electric field, the upward polarization state can also be formed at the same area. Thus, the existence of two well-defined ferroelectric polarization states, as well as their switchability for both film regions, can be again confirmed.

In addition, we observed that the downward polarization orientation is stable and preferential. And the upward ferroelectric state shows a remarkable polarization relaxation effect, which is predominantly ascribed to the depolarization field and the built-in electric field.^44–46^ These results are consistent well with that of our previous report.^32^ To further illustrate the temperature dependence of local polarization nature for both regions, we thus estimated the value of the polarization relaxation time as the period of which before the upward polarization relaxed switch to the downward polarization. Table 1 summarized the polarization relaxation time for both the grain and intergrain regions at four different temperatures. In the case of the grain region, a much improved polarization relaxation time of 6200 ms can be observed when the temperature is increased from room temperature to 40 °C. And the polarization relaxation time decreases with further increase in the annealing temperature. Besides, we also obtained a similar evolution of the polarization relaxation on the intergrain regions. At 40 °C, the value of the polarization relaxation time can reach up to 3400 ms, which is one to three magnitude of order higher than that of other temperatures. These results indicate that the optimum local ferroelectric characteristics for both film regions are also achievable when the ultrathin films are post-annealed at 40 °C. Additionally, the optimal polarization relaxation time can be reasonably ascribed to a highly packing order of the polymer chain of ultrathin crystalline P(VDF–TrFE). In our separate study, we also discovered that the bi-stable ferroelectric polarization of ultrathin P(VDF–TrFE) can be obtained on the Pt and semi-In_2_O_3_ substrates, which is greatly appealing for high-density data storage and ferroelectric tunnel junctions. The further study on the intrinsic mechanism is being carried out (Fig. S8†).

**Table 1:** The relationship between the polarization relaxation time, film region, and temperature

Polarization relaxation (ms)				
Region	Temperature (°C)			
	Room temp	40	50	60
Grain region	820	6200	75	62
Intergrain region	100	3400	50	8

Considering that P(VDF–TrFE) is an intrinsically semi-crystalline polymer, whose film is formed basically with the alternating crystalline and amorphous slabs.^33,37^ Thus, the fact that the intergrain region exhibits local piezo- and ferroelectric responses (as shown in Fig. 5–7 above) can exclude the effect of pinholes. According to the previous study, the partial crystallinity of the intergrain region can be confirmed.^47^ And a similar evolution of *d*_*zz*_ of the intergrain region compared with the grain region indicates that the crystalline structure of the intergrain region is presumably related to that of the lamellae grain (Fig. 6). Hence, it is reasonable to speculate that the crystallites inside this region are randomly oriented chain segments and tie molecules, whose sizes are too small to be clearly resolved *via* the AFM characterization. Therefore, the obtained PFM hysteretic behaviours proved that the ferroelectric polarization reversal should occur when applying an external poling voltage for both the grain and intergrain regions. However, the orientation changing of the dipoles had distinguishing energy barriers for two regions, thus resulting in different coercive voltages. For the grain region, an identical barrier should be overcome by various electric dipoles for polarization reversal because of the ordered arrangement of the polymer lattice structure. Thus, an abrupt polarization switching was presented. Besides, the intergrain region was composed of small semi-ordered crystallites and amorphous matrix. It meant that various electric dipoles were surrounded by different microscopic environments, that is, different energy barriers were needed to undergo during the polarization reversal for different dipoles. This difference therefore fully explained the broadening of the coercive voltage distribution. Additionally, the intergrain region can demonstrate a considerable piezoresponse in our ultrathin P(VDF–TrFE) films. It most probably arises from a predominated dipole-induced electrostriction of the crystalline chain segments in tandem with an additional electromechanical coupling contribution between the amorphous part and polar crystallites.^37^ Moreover, such a discrepancy of local piezoresponse between different regions has also been reported in Langmuir–Blodgett P(VDF–TrFE) films.^48^

Furthermore, the dipole value of the α-chain is comparable to that of the β-phase for P(VDF–TrFE), and thus the orientation degree of the electric dipoles and polymer chains is highly critical for the piezo- and ferroelectric properties of P(VDF–TrFE) films.^10,34^ The height of the specific GI-XRD (*h k* 0) peak can be used to elucidate the mainchain packing degree.^10,21,49–51^ As shown in Fig. 3 and S8,† the strongest intensity of the (110) GIXRD diffraction peak can be obtained at 40 °C, indicating that a more highly ordered packing of the polymer chains in the film plane relative to that of other temperatures. Note that the molecular electric dipoles are oriented perpendicular in relation to the polymer chain plane, thus the electrical polarization relies on the angle between the external poling field and polymer backbone.^50^ Therefore, when most polymer chains preferably orient normal to the applied poling field, it ensures a greatly effective contribution to the ferroelectric polarization during a local dipole rotation process.^10^ Previous theoretical study has also shown that the electrical polarization can be significantly enhanced under such a highly orientation degree.^52^ As discussed above, the optimum piezo- and ferroelectric characteristics obtained at 40 °C can be attributed to a notably ordered orientation of the polymer chains and molecular dipoles.

As a special geometry confinement system, although the packing order and orientation of ultrathin polymer chains are largely altered by complicated interfacial enthalpic and entropic effects, whose intrinsic physical mechanisms are under intensive debate.^53,54^ The optimal piezo- and ferroelectricity of 40 °C-annealed films can be considered to be originated from a combining effect, which includes the thermally induced lattice reorganization and dipole interaction.^21,22,55^ On applying a 40 °C annealing treatment, the nucleation and growth of lamellar crystals should occur along an energetically preferential specific (110) plane.^22^ Another issue deserving great attention is the dipole interaction, a dominating drive force for the size effect in molecular ferroelectrics.^21^ When an available conformation of a crystalline P(VDF–TrFE) obeys a strong geometrical constraint, the temperature can exert a profound influence on the dipole orientation and crystalline structure, as revealed by our results. Additionally, the polymer lattice orientation changes under different temperature can be attributed to the competition between the chain attachment and detachment associated with the configuration and mobility of the polymer chain at the nanoscale.^22,56^ Moreover, the non-180° phase contrast and small piezoelectric strain at 50 and 60 °C can be mainly governed by a combined effect of the nearly lying-down dipole orientation and severe polarization relaxation.

## Conclusions

In summary, the morphologies and concomitant structure changes of quasi-2D ultrathin polycrystalline P(VDF–TrFE) films annealed at different temperatures were observed. More importantly, the temperature-dependent piezo- and ferroelectric properties were unambiguously demonstrated by measuring the polarization-patterned domains, local PFM hysteretic loops, local piezoelectric coefficients *d*_*zz*_, and ferroelectric polarization relaxation. In particular, the optimal film properties can be obtained *via* a post-annealing treatment at 40 °C, which originated from a high polymer chain arrangement degree. This result made our ultrathin P(VDF–TrFE) film a competitive candidate for promising nano-electro-mechanical systems (NEMS) and ultrahigh-density data storage applications.

## Conflicts of interest

The authors declare no conflict of interest.

## Supplementary Material

RA-008-C8RA05648J-s001
