# Supplementary material for: Temperature dependence of piezo- and ferroelectricity in ultrathin P(VDF–TrFE) films
Source: RSC Adv. 2018 Aug 16;8(51):29164–71. doi: 10.1039/c8ra05648j (PMC9084441; doi:10.1039/c8ra05648j)
Supplement: RA-008-C8RA05648J-s001 [file RA-008-C8RA05648J-s001.pdf]

Electronic Supplementary Material (ESI) for RSC Advances.
This journal is © The Royal Society of Chemistry 2018

Supporting information for "Temperature dependence of piezo- and ferroelectricity in ultrathin P(VDF-TrFE) films"

Jun Qian, Sai Jiang, Qijing Wang, Chengdong Yang, Yiwei Duan, Hengyuan Wang, Jianhang Guo, Yi Shi and Yun Li*

National Laboratory of Solid-State Microstructures, School of Electronic Science and Engineering, Collaborative Innovation Center of Advanced Microstructures, Nanjing University, Nanjing 210093, P. R. China. Email: yli@nju.edu.cn.

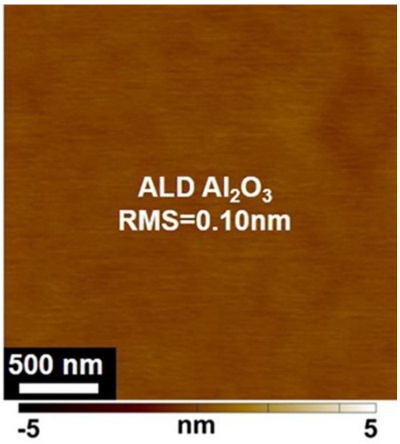

Fig. S1 The AFM morphological image of the ALD Al_2O_3 substrate.

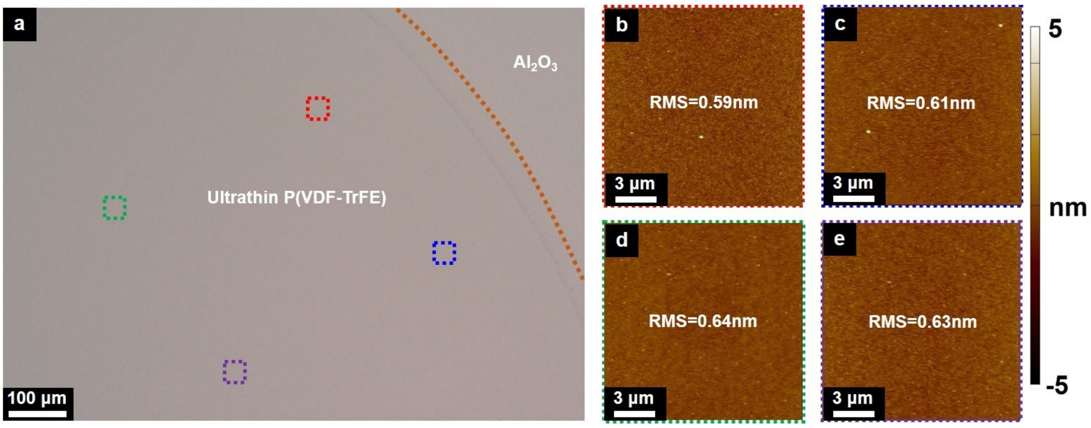

Fig. S2 The ultrathin crystalline P(VDF-TrFE) on the Al_2O_3 substrates. As shown in the Fig. S2a, the solution-coated P(VDF-TrFE) film is extremely uniform over a large area of ~1.1 mm. AFM topographical images were taken from randomly chosen 4 areas as marked in the optical microscopy image (Fig. 2a), ultrathin films of crystalline P(VDF-TrFE), exhibit a root-mean-squared (r.m.s.) roughness of less than 1 nm. The ultrasmooth films can be potentially beneficial for a small leakage current and thus a good reliability of piezo- and ferroelectric functional elements.

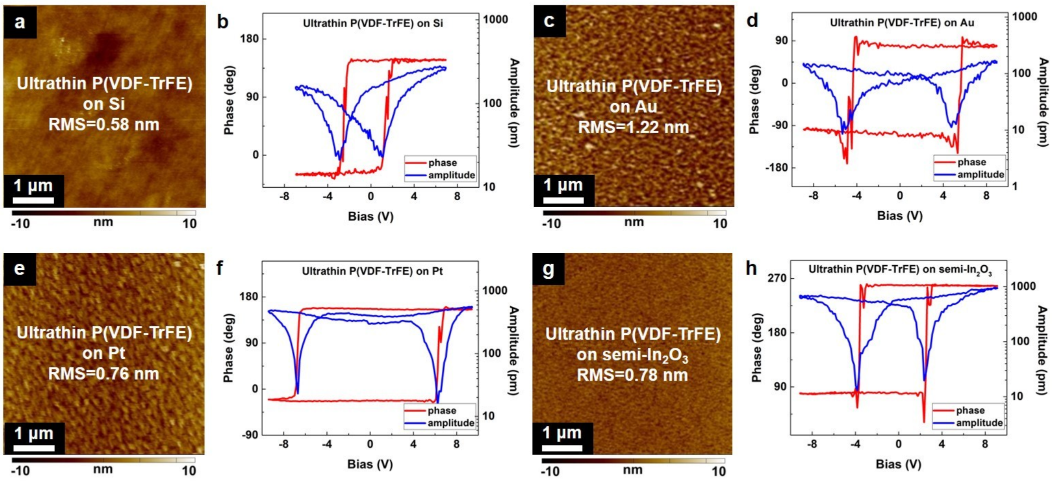

Fig. S3 The ultrathin crystalline P(VDF-TrFE) on the different substrates, such as silicon, Au, Pt, and semiconducting In_2O_3.

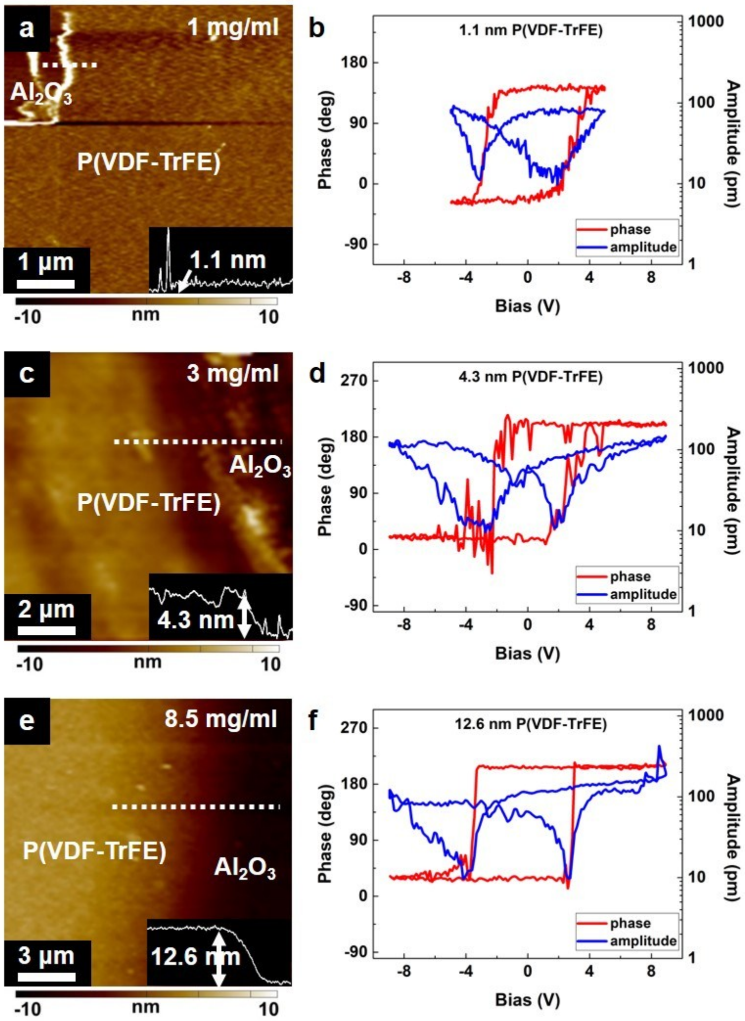

Fig. S4 The ultrathin crystalline P(VDF-TrFE) with different thicknesses, which can be easily tuned by the P(VDF-TrFE) concentration in the solution.

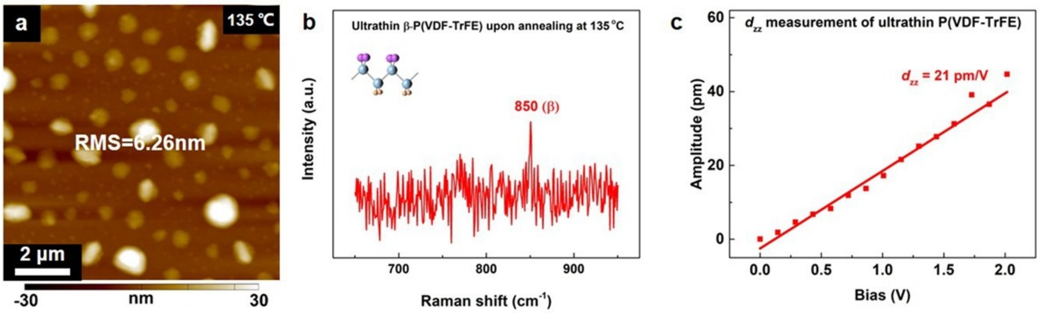

Fig. S5 (a) The AFM morphological image of an ultrathin P(VDF-TrFE) film achieved by an antisolvent-assisted-crystallization technique and annealing at 135 ℃ for 10 min. (b) The Raman spectra of the film shown in (a). (c) local piezoelectric coefficient d_{zz} measurement of the ultrathin P(VDF-TrFE).

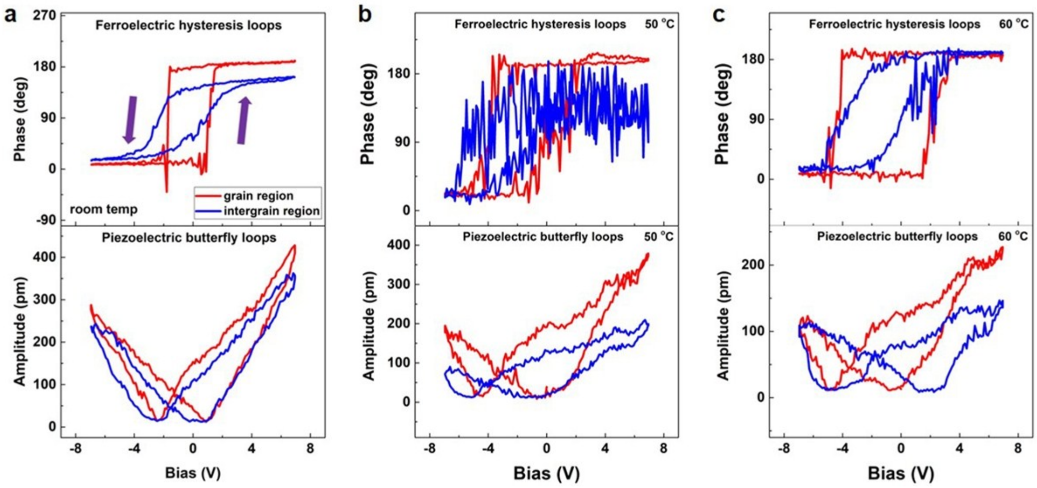

Fig. S6 Local PFM hysteresis loops of ultrathin P(VDF-TrFE) films with different annealing temperatures. The arrows indicate the sweep direction.

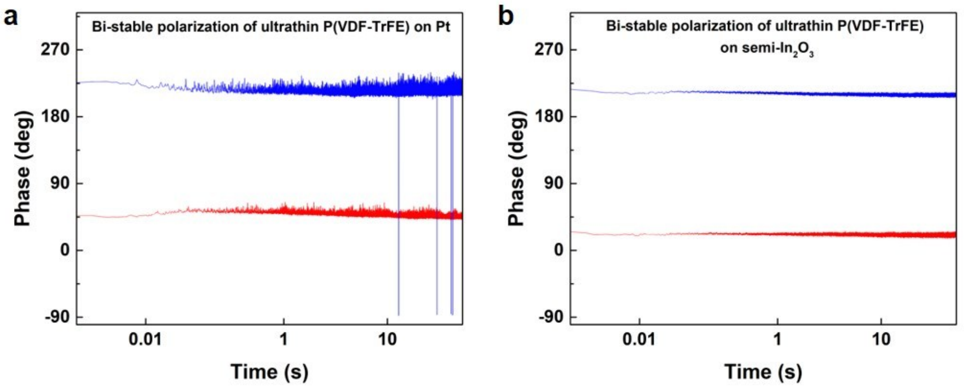

Fig. S7 Bi-stable polarization of ultrathin P(VDF-TrFE) films on Pt and semi-In_2O_3 substrates, respectively.

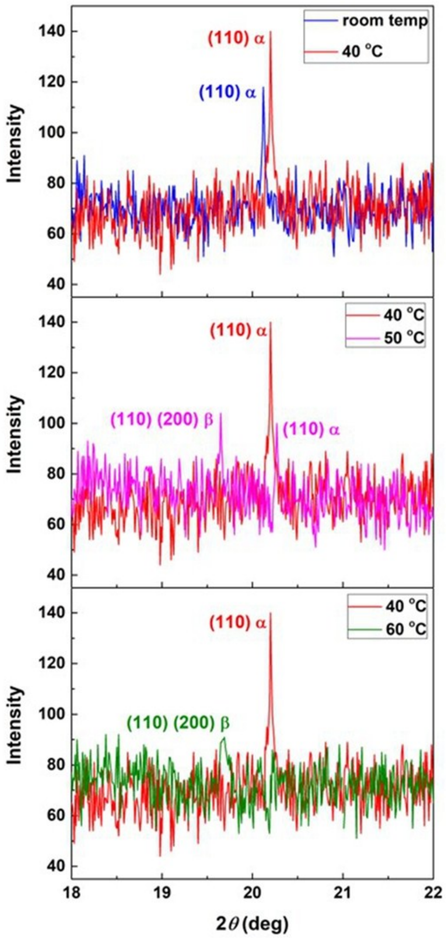

Fig. S8 The comparison of GIXRD (*h k* 0) peak intensity between 8.2 nm-thick crystalline P(VDF-TrFE) films at four different temperature levels.
